# Potential Orphan Drug Therapy of Intravesical Liposomal Onabotulinumtoxin-A for Ketamine-Induced Cystitis by Mucosal Protection and Anti-inflammation in a Rat Model

**DOI:** 10.1038/s41598-018-24239-9

**Published:** 2018-04-11

**Authors:** Wei-Chia Lee, Chia-Hao Su, You-Lin Tain, Cheng-Nan Tsai, Chun-Chieh Yu, Yao-Chi Chuang

**Affiliations:** 1 Division of Urology, Kaohsiung Chang Gung Memorial Hospital and Chang Gung University College of Medicine, Kaohsiung, Taiwan; 2 Institute for Translational Research in Biomedicine, Kaohsiung Chang Gung Memorial Hospital, Kaohsiung, Taiwan; 3 Department of Pediatrics, Kaohsiung Chang Gung Memorial Hospital and Chang Gung University College of Medicine, Kaohsiung, Taiwan; 4 Center for Shock Wave Medicine and Tissue Engineering, Kaohsiung Chang Gung Memorial Hospital and Chang Gung University College of Medicine, Kaohsiung, Taiwan

## Abstract

Ketamine abusers may develop ulcerative cystitis and severe lower urinary tract symptoms, which is a medical dilemma. Recently, researchers have found the endemic of ketamine-induced cystitis worldwide. The intravesical administration of liposome-encapsulated onabotulinumtoxinA (Lipotoxin) might facilitate the healing of the damaged urothelium from liposomes, and reduce the urinary symptoms by onabotulinumtoxinA-induced chemo-denervation. Using female Sprague-Dawley rats, we investigated the effects of Lipotoxin on ketamine-induced cystitis. Functional magnetic resonance imaging, metabolic cage study, and cystometry were conducted. Paraffin-embedded sections were stained. The bladder mucosa and muscle proteins were assessed through Western blotting. We observed that repeated intravesical Lipotoxin instillation could improve suburothelial hemorrhage, recover the urothelial tight junction and adhesion proteins (zonula occludens-1 and E-cadherin), ensure less substance P in the urothelium, inhibit the overexpression of inflammatory mediators (IL-6, TNF-α, nuclear NF-κB, and COX-2) in the detrusor, suppress the upregulation of the mucosal TRPV1 and detrusor M_2_-mAChR, and ameliorate bladder overactivity in the ketamine-treated rats. These data reveal the mechanisms underlying the action of Lipotoxin in ketamine-induced cystitis of rats, which provide a basis of Lipotoxin for further treating ketamine-induced cystitis in humans.

## Introduction

Ketamine-induced cystitis (KIC) is characterized by its interstitial cystitis-like symptoms among ketamine abusers, namely a painful bladder, dysuria, urgency, urinary frequency, and hematuria^1^. The grossly pathological findings of KIC are a contracted bladder and ulcerative cystitis^2^. The use of ketamine as a recreational drug has consistently increased worldwide in the past few decades^1,3^. For instance, the amount of ketamine seized in drug arrests in Taiwan increased from 799.5 kg in 2008 to 2393.2 kg to 2013^2^. Moreover, the discovery of the rapid antidepressant effect of ketamine and its metabolites could lead to the situation of off-label use and drug abuse worse^4,5^. The management of patients with KIC is challenging for urologists, and abstinence from ketamine is the first step to managing such patients^2^. However, urinary symptoms may persist up to 1 year or more even after the cessation of ketamine use^6^. A few of therapeutic strategies were reported in selective cases^2,3^, such as intravesical instillation of hyaluronic acid, and intradetrusor injection of botulinum toxin A (BoNT-A); however, both strategies often failed to yield therapeutic effects. Augmentation enterocystoplasty is the final with certain complications^7^. The entity of KIC remains unclear. Researchers have established animal models of KIC^8–10^ to understand its pathophysiology and develop novel treatments. They reported that urothelial barrier dysfunction, neurogenic inflammation, and aberrant bladder neurotransmission are the main pathologies of KIC.

Liposome-encapsulated onabotulinumtoxinA (Lipotoxin) is a novel bladder instillation therapy for treating overactive bladder or interstitial cystitis in clinical trails^11–13^. Liposomes have vesicular structures comprising an aqueous core surrounded by a lipid bilayer, which allows the delivery of hydrophilic BoNT-A across the urothelium through endocytosis without injections^11,14^. BoNT-A inhibits vesicular neurotransmitter release at the neuromuscular and neuroglandular junctions by cleaving the synaptosomal-associated protein, 25 kDa (SNAP25) responsible for the exocytosis of synaptic vesicles^15^. Through this mechanism of action, BoNT-A can cause motor effects on partial paralysis of detrusor and sensory effects on inhibition of afferent neurotransmission by blocking release of sensory neurotransmitters, such as substance P and calcitonin gene-related peptide. Therefore, BoNT-A can modulate aberrant neurotransmission and ameliorate bladder inflammation. The US Food and Drug Administration approved cystoscopic BoNT-A injection in the treatment of idiopathic overactive bladder and neurogenic detrusor overactivity^15^. Moreover, American Urology Association recommends intradetrusor BoNT-A injection for intractable interstitial cystitis^15^. Empty liposomes alone can provide coating and healing effects on damaged urothelium of interstitial cystitis patients^16^. Hence, the potential activity sites for the intravesical instillation of Lipotoxin include the urothelium and vesical afferent and efferent signal pathways^11^.

In this translational study, we investigate the potential of intravesical Lipotoxin instillation for treating KIC in a rat model^9^. The alterations of micturition behavior, urothelial injuries, and inflammatory mediators and neuroreceptors of the bladder were evaluated.

## Results

### General characteristics, functional magnetic resonance imaging, metabolic cage study, and cystometry

**Figure 1:**
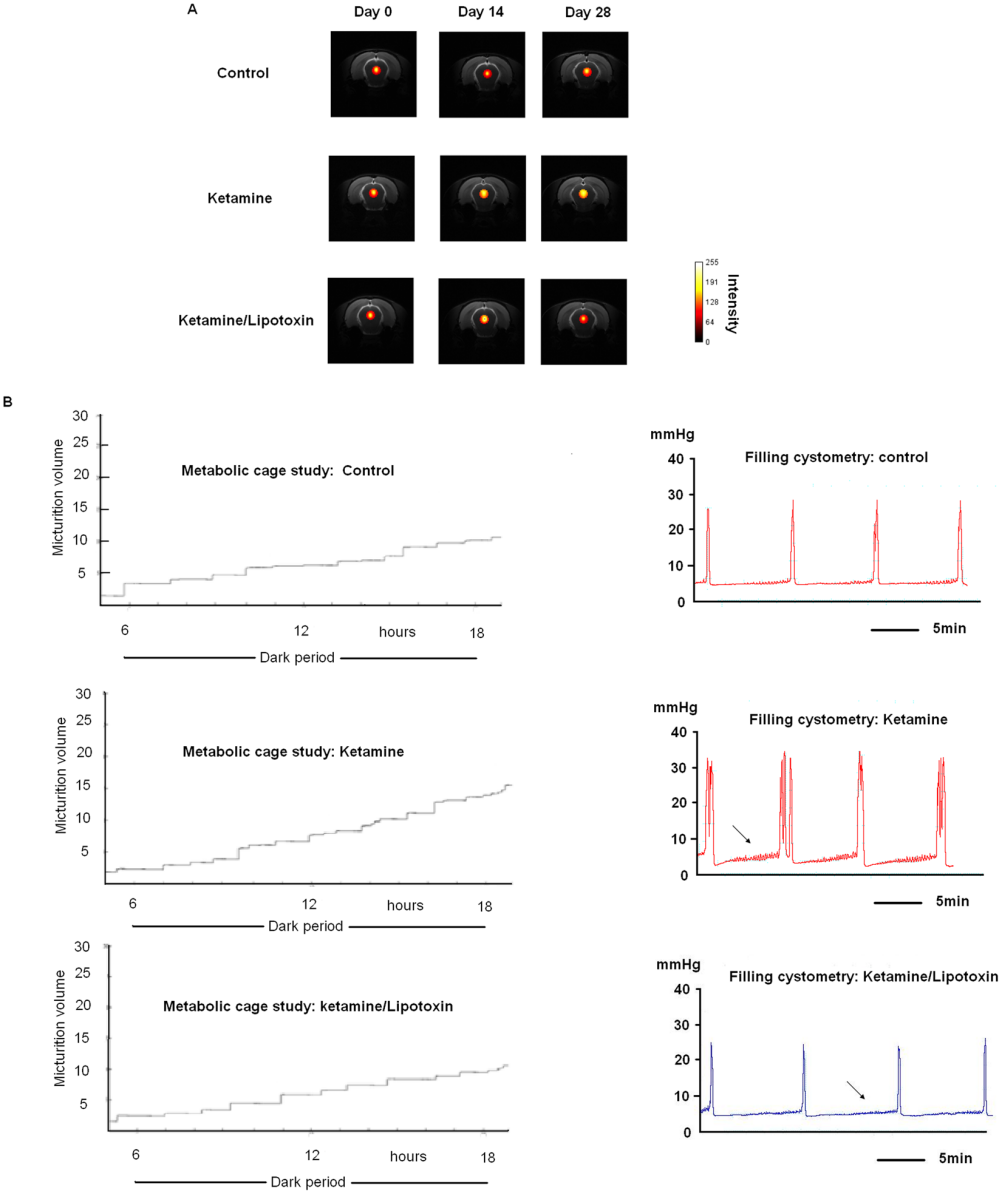
Micturition behavior evaluation of rats. (**A**) T2-weighted image on brain coronal sections of rats with empty bladders in fMRI. Hyperactivated signals were observed in the PAG region of ketamine-treated rats on days 14 and 28, but not in the Lipotoxin-treated rat on day 28. (**B**) Representative traces of conscious metabolic cage study and anesthetized cystometry. Increased micturition frequency and shorten intercontractile intervals were observed in the ketamine group. The arrows indicate the increased basal tone of ketamine-treated rats and subsided with Lipotoxin treatment.

Table 1 and Fig. 1 present the general characteristics and micturition behavior of rats in all groups. The concentrations of ketamine and norketamine in the urine of rats in the ketamine and ketamine/Lipotoxin groups were markedly high, in contrast to the undetectable concentrations in the control group. Compared with control group, the ketamine and ketamine/Lipotoxin groups showed significantly increased mean bladder weight. We observed hyperintense functional magnetic resonance imaging (fMRI) signals in the periaqueductal grey (PAG) area (Fig. 1A), increased micturition frequency (Fig. 1B) in the metabolic cage study, and reduced cystometric intercontractile intervals in the ketamine group. However, no significant difference was observed between the ketamine/Lipotoxin and control groups in the MRI signals in the PAG area (day 28), micturition frequency, and intercontractile intervals.

**Table 1:** General characteristics and micturition behavior of experimental animals, n = 12 in each group.

	Mean ± SEM		
	Controls	Ketamine	Ketamine/Lipotoxin
**General characteristics:**			
Body wt (gm), day 1	225.4 ± 1.6	221.1 ± 3.1	218.0 ± 1.8
Body Wt (gm), day 28	273.4 ± 4.1	264.2 ± 7	263.7 ± 5.5
Difference of body wt (gm)	48.0 ± 4	43.2 ± 5	45.7 ± 4.6
Bladder wt (mg)	96.3 ± 5.2	118.6 ± 5.1*	117.8 ± 5.1*
Bladder wt/Body wt (mg/g)	0.35 ± 0.02	0.45 ± 0.02*	0.45 ± 0.02*
**Metabolic cage study/24 h**, **day28**			
No. Voids	18.1 ± 0.8	22.8 ± 1*	18.6 ± 0.9
Water intake (ml)	35.5 ± 1.9	34.2 ± 0.9	37.9 ± 1
Urine output (ml)	19.7 ± 1	16.8 ± 1.3	19.5 ± 0.7
**Cystometry parameter**			
Voiding pressure (mmHg)	30.3 ±± 1.1	35.1 ± 2.6	30.9 ± 2.2
Intercontractile interval (min) 16.7 ± 2.2	11.1 ± 0.6*	15.5 ± 0.8	
**Urine parameters**			
Urine ketamine (ng/ml)	ND	2036 ± 302.8*	1615 ± 508.9*
Urine norketamine (ng/ml)	ND	16826 ± 2232.1*	11697 ± 4577.7*

ND, not detected.

*Dunnett’s test showed a significant difference between controls and the other groups.

### Mucosal protection and chemical denervation by Lipotoxin instillation, as revealed by histology and Western blotting

**Figure 2:**
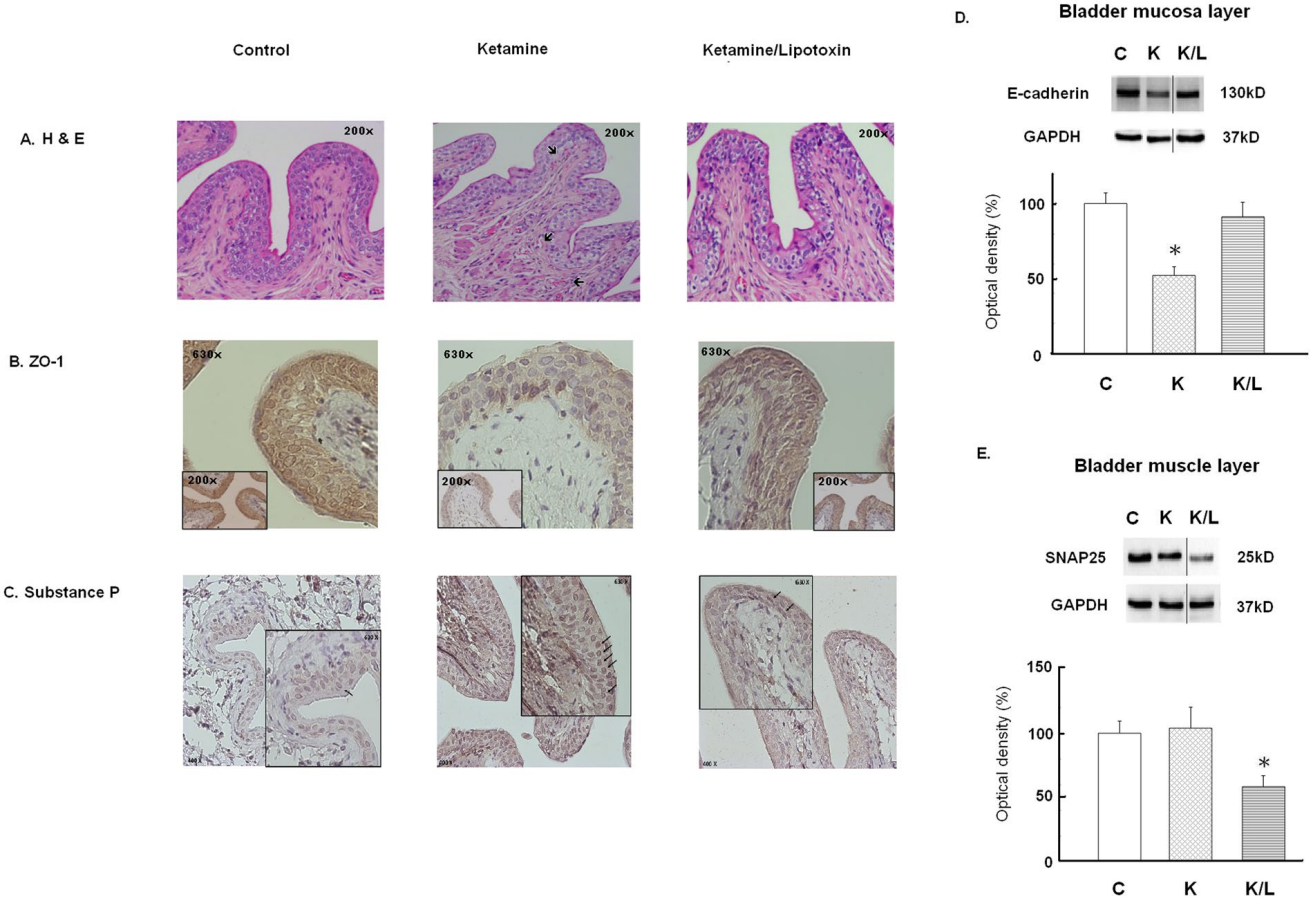
Mucosal protection and chemical denervation effects of Lipotoxin on the ketamine-treated rats’ bladders. (**A**) Red blood cell debris under suburothelium of rats in the ketamine group (arrows). H & E, reduced from ×200. (**B**) Faint immunostaining of ZO-1 on the urothelium of ketamine-treated rats, and much improvement with Lipotoxin treatment. Reduced from ×200 and ×630. (**C**) Immunostaining of substance P. The abundant substance P stains were found on the rat’s urothelium of the ketamine group. The peppercorn-like spots indicate the substance P staining (arrows). Reduced from ×200 and ×630. (**D** and **E**) Western blots of mucosal E-cadherin and detrusor SNAP25. Data are expressed as means ± SEM. n = 8. *p < 0.05 versus controls. The grouping of blots was cropped from the same gel for each protein. The full-length gels and blots are included in the Supplementary Figure S2.

Figure 2 shows the histological features of ketamine-associated bladder damage and effects of bladder Lipotoxin instillation. Compared to controls, the ketamine group showed markedly high red blood cell debris under the suburothelium, faint immunolabeling of the zonula occludens (ZO)-1 (tight junction protein), significantly reduced expression of E-cadherin (adhesion protein), and enhanced immunolabeling of substance P in urothelium. In contrast, the ketamine/Lipotoxin group did not. Moreover, the ketamine/Lipotoxin group showed reduced SNAP25 expression in the detrusor, indicating BoNT-A action.

### Response of inflammatory mediators and neuroceptors to ketamine insults and Lipotoxin treatment

**Figure 3:**
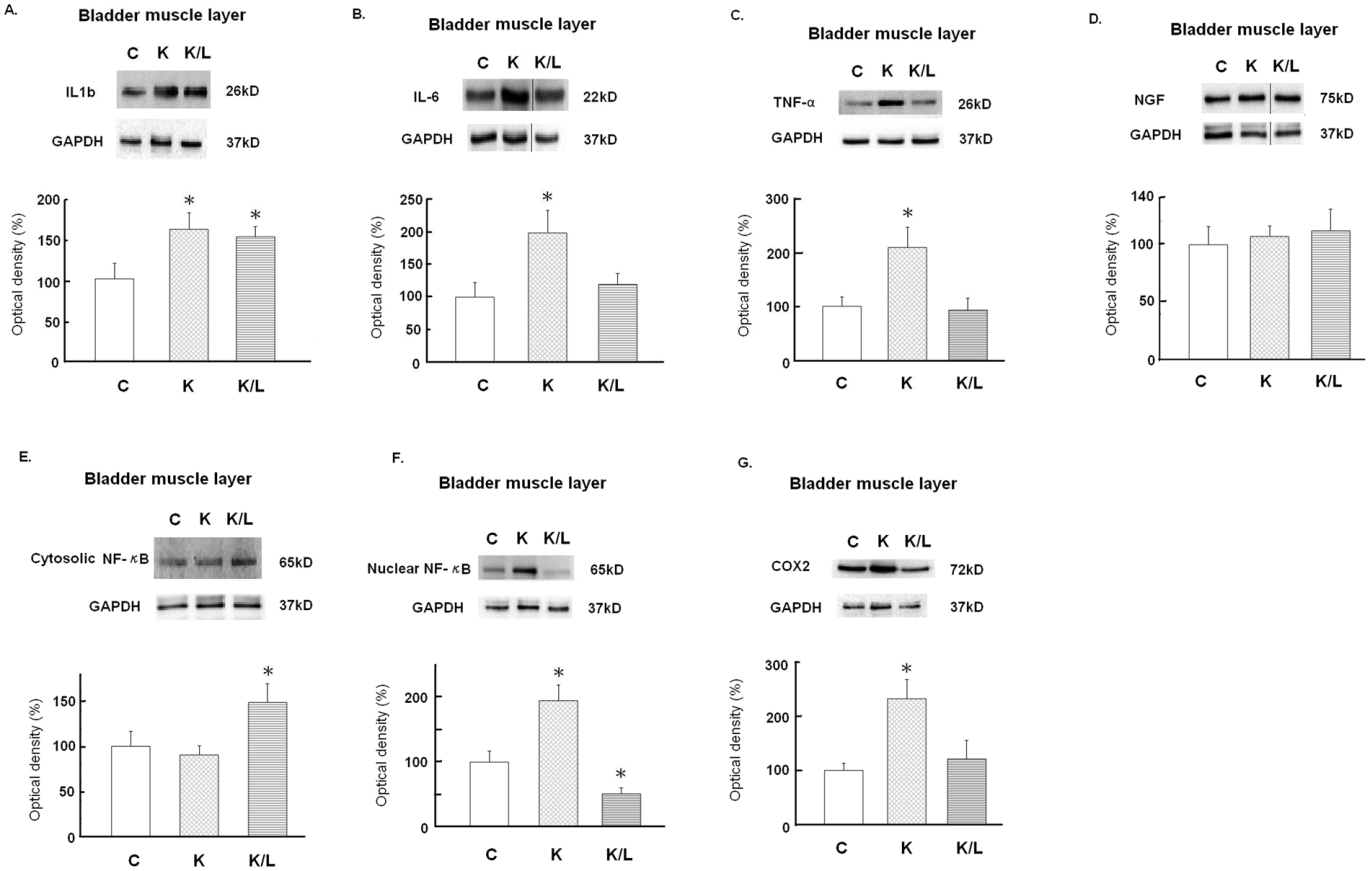
Anti-inflammatory actions of Lipotoxin on ketamine-treated rats’ bladders. Western blot analyses with specific antibodies to inflammatory mediators of rat detrusor samples were performed for all groups (n = 8). (**A**) IL-1β, (**B**) IL-6, (**C**) TNF-α. (**D**) Nerve growth factor. (**E**) Cytosolic NF-κB. (**F**) Nuclear NF-κB. (**G**) COX2. Experiments were repeated two times and representative blots are shown (upper). Data of proteins expression (ratios of signal intensities of investigated proteins relative to GAPDH) were calculated with 8 samples in each group. These data of Mean ± SE were standardized and expressed in percentage in which the value of the control group is treated as 100%. Theses values were shown in the bar graph (lower). An asterisk indicates a significant difference between controls and other groups (One-way ANOVA with Dunnett’s test, p < 0.05). The grouping of blots was cropped from the same gel for each protein. The full-length gels and blots are included in the Supplementary Figure S3.

**Figure 4:**
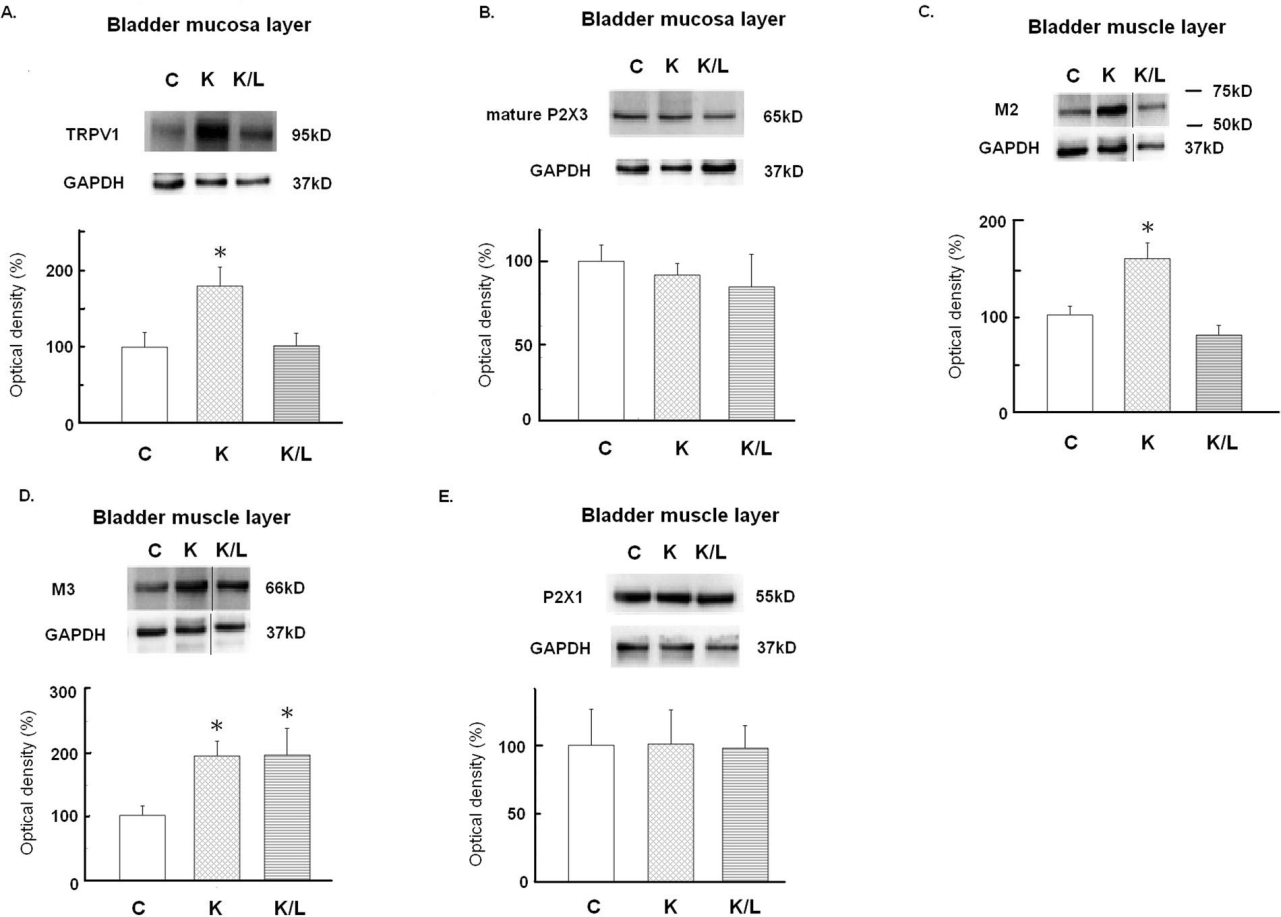
Alterations of neuroreceptors protein expression in the mucosa layer or smooth muscle layer of the bladder for all groups (n = 8). Western blot analyses with specific antibodies to the TRPV1 receptor and P2X_3_ receptor of the rat mucosal layer as well as M_2_-and M_3_- mAChRs and the purinergic P2X_1_ receptor of the rat detrusor layer were performed in 3 groups. (**A**) TRPV1 receptor: The TRPV1 antibody produced a clear single band at 95 kDa. (**B**) Purinergic P2X_3_ mature receptor: the predominant P2X_3_ form (65 kDa). (**C**) M_2_ –mAChR of bladder detrusor layer. The M_2_ –mAChR antibody produced a clear single band between 50kD and 75kD. (**D**) M_3_ –mAChR of bladder detrusor layer. (**E**) Purinergic P2X_1_ receptor. Experiments were repeated two times and representative blots are shown. Data of proteins expression (ratios of signal intensities of investigated receptors relative to GAPDH) were calculated with 8 samples in each group. These data of Mean ± SE were standardized and expressed in percentage in which the value of the control group is treated as 100%. Theses values were shown in the bar graph. An asterisk indicates a significant difference between controls and other groups (One-way ANOVA with Dunnett’s test, p < 0.05). The grouping of blots was cropped from the same gel for each protein. The full-length gels and blots are included in the Supplementary Figure S4.

Figures 3 and 4 present representative results of Western blotting and statistical comparisons of protein expression in the bladder mucosa and detrusor of the three groups. Compared with the control group, the ketamine group showed significant overexpression of the inflammatory mediators in the detrusor, including interleukin (IL)-1β, IL-6, tumor necrosis factor (TNF)-α, nuclear nuclear factor (NF)-κB, and cyclooxygenase (COX)-2, as well as neuroreceptors in the bladder mucosal transient receptor potential vanilloid 1 (TRPV1) receptor, and M_2_, and M_3_- muscarinic acetylcholine receptors (mAChRs) in the detrusor. However, the ketamine/Lipotoxin group showed no significant differences compared with the control, except for higher IL-1β and M_3_-mAChR expression levels in the detrusor.

## Discussion

Our findings reveal that repeated intravesical Lipotoxin instillation can ameliorate KIC in rats. Lipotoxin instillation can provide the dual effects of mucosal protection and chemical denervation for treating ketamine-induced micturition symptoms and bladder overactivity, mucosal damage, and inflammation. Compared with the control group, the ketamine group showed a stronger activation of the PAG area in fMRI, despite an empty bladder, and significantly increased micturition frequency and cystometric bladder overactivity; however, the ketamine/Lipotoxin group did not. Daily ketamine injection caused suburothelial hemorrhage, reduced urothelial tight junction (ZO-1) and adhesion protein (E-cadherin), and enhanced substance P spread over the urothelium in the rat bladders. The ketamine/Lipotoxin group exhibited sparse suburothelial hemorrhage, improved mucosal tight junction and adhesion protein loss, and less substance P spread in the urothelium, in addition to exhibiting decreased SNAP25 in the detrusor. Furthermore, the mechanisms underlying the action of Lipotoxin in treating KIC may involve the modulation of inflammatory mediators and neuroceptors in rat bladders, and disruption of central sensitization, as revealed by reduced fMRI signals in the brain. The ketamine group had significantly increased inflammatory mediators, namely IL-1β, IL-6, TNF-α, nuclear NF-κB, and COX-2, in the detrusor. Moreover, this group showed significant overexpression of bladder mucosal TRPV1 receptor, and detrusor M_2-_and M_3_-mAChRs. However, the ketamine/Lipotoxin group had insignificant changes from controls, except for IL-1β and M_3_-mAChR overexpression in the detrusor. These results demonstrate that repeated Lipotoxin instillation may have the potential in treating KIC by ameliorating bladder overactivity, ensuring mucosal protection, inhibiting detrusor inflammation, modulating the aberrant vesical neurotransmission, and inhibiting central sensitization.

KIC is notorious for its severe urinary symptoms and ulcerative cystitis^2^. At present, the best KIC animal model available was established by Chuang *et al*.^9,17,18^, representing the micturition behavior in and bladder pathology^2,19^. In the current study, we observed that the bladder instillation of Lipotoxin reduced vesical afferent inputs in the PAG area, micturition frequency and bladder overactivity in ketamine-treated rats. These beneficial effects might be a result of the Lipotoxin-induced dual effects of mucosal protection and chemical denervation^11^. Micturition behavior involves the interaction of the bladder with the central nervous system. The neural circuitry in supraspinal control system, particularly in the PAG area, plays an important role in the occurrence of urgency^20,21^. The vesical afferent signals emerge into the PAG area. The PAG area signals can enable understanding the peripheral sensory input from the bladder and be a reference of “afferent noise” intensity^21,22^. In the current study, the Lipotoxin-induced inhibition of central sensitization might provide evidence demonstrating that intravesical Lipotoxin instillation can abate bladder afferent noise caused by KIC insults and avoid the possible neural plasticity in urgent sensation^23^.

Repeated liposomes instillation may result in mucosal protection effects in ketamine-treated rat bladders. Our results reveal sparse suburothelial hemorrhage, improved urothelial tight junction and adhesion protein damage, and less substance P spread in the bladder of rats in the ketamine/Lipotoxin group. Liposomes have been reported to protect the bladder from irritating urine solutes and promote the healing of urothelial damage^24–26^. Tyagi *et al*. reported that liposomes instilled in a rat’s bladder formed a coating on the urothelium surface and blocked the irritation of the submucosal afferents^24^. Thus, this liposome coating may cover the injured urothelium and block the direct toxic damage induced by ketamine and its metabolites on the bladder of ketamine/Lipotoxin-treated rats. Reduced submucosal afferent excitation after liposomes instillation might be reflected in the less spread of substance P over the urothelium in this study. Certainly, C-fiber denervation caused by BoNT-A may contribute to this phenomenon in Lipotoxin-treated rats^11,15^. Chuang *et al*.^9^ reported that daily ketamine injection could cause denuded urothelium and suburothelial hemorrhage in rats on day 14. Our fMRI results reveal stronger activation of the PAG area in ketamine-treated rats on day 14. However, less bladder mucosal damage was observed in the ketamine/Lipotoxin group on day 28. We believe that liposomes account for the direct repair of bladder urothelial deficits, such as the recovery of the tight junction and adhesion protein in the ketamine/Lipotoxin group.

Our study supports the notion that bladder instillation of Lipotoxin can deliver BoNT-A into rats’ detrusor and cause chemo-denervation, which modulates the inflammatory mediators and aberrant vesical neuroreceptors of chemical cystitis in rats^11^. In this study, detrusor SNAP25 decreased in the ketamine/Lipotoxin group. Meanwhile, Lipotoxin suppressed the upregulation of bladder mucosal TRPV1 receptor and detrusor M_2_-mAChR, and inhibited the overexpression of IL-6, TNFα, COX-2 and nuclear NF-κB proteins in the detrusor of ketamine-treated rats. Direct toxic damage caused by ketamine and its metabolites is considered to induce a cascade of bladder pathology of KIC, namely bladder barrier dysfunction, neurogenic inflammation, and COX-mediated inflammation^2^. After toxic substances activate vesical C-fibers, substance P release plays a key role in inflammatory response initiation^27^. Juan *et al*. reported that NF-κB translocation into the nucleus could activate COX-2 mediated inflammation and fibrosis of KIC^17^. Through Lipotoxin instillation and its action on C-fiber denervation^11,15^, BoNT-A may suppress mucosal TRPV1 receptor upregulation and prevent substance P release in ketamine-treated rat bladders. Through such mechanisms, Lipotoxin could inhibit the neurogenic inflammation and modulate a series of inflammatory mediators, such as IL-6, TNF-α, nuclear NF-κB and COX-2 in the detrusor of rats in the ketamine/Lipotoxin group. Moreover, the cholinergic denervation effect of BoNT-A can suppress the upregulation of detrusor M_2_-mAChR in ketamine/Lipotoxin-treated rats, as our results and a previous report^15^. Thus, Lipotoxin treatment can improve the aberrant neurotransmission and associated inflammatory response of KIC.

This is the first report of the promise of Lipotoxin in KIC treatment; however, the study has some limitations. This study did not include a control group of liposomes administration alone, which would limit us to speculate on the added effect of botulinum toxin and the mechanisms as to how lipotoxin is causing changes in both mucosa and smooth muscle layer. Although we demonstrated the therapeutic effects of Lipotoxin in a rat model of KIC, the results cannot be completely generalized to humans. Furthermore, the dose-effect relationship should be determined before we can apply Lipotoxin for human treatment.

## Conclusion

Our study demonstrates that repeated Lipotoxin bladder instillation can ameliorate the ketamine-induced bladder overactivity and chronic inflammation in rats by inducing mucosal protection and chemical denervation. Furthermore, repeated Lipotoxin treatment could improve suburothelial hemorrhage, recover urothelial tight junction and adhesion proteins, ensure less substance P in the urothelium, inhibit the overexpression of inflammatory mediators (IL-6, TNF-α, nuclear NF-κB, and COX-2) in the detrusor, and suppress the upregulation of the mucosal TRPV1 and detrusor M_2_-mAChR in the ketamine group. Our results reveal the mechanisms underlying the action of Lipotoxin in KIC, which can facilitate future clinical trials on an orphan drug.

## Materials and Methods

This study was conducted in accordance with the guidelines of National Research Council, USA, and the Animal Protection Law by the Council of Agriculture of the Republic of China. The experimental protocol was approved by the Institutional Animal Ethics Committee of Chang Gung Memorial Hospital (permit number: 2014121104). Invasive procedures were performed under anesthesia, and every effort was made to minimize both the suffering and number of animals used in the experiments.

Sixty female Sprague-Dawley rats (BioLASCO Taiwan Co., Ltd., Taipei, Taiwan; weight: 200–250 g) were randomly allocated to three groups (n = 20) and subjected to an experimental course of 28 days. They were maintained in a facility accredited by the Association for Assessment and Accreditation of Laboratory Animal Care International under temperature control (24 ± 0.5 °C) and a 12:12-h light-dark cycle. The groups were as follows: i) a control group (0.9% saline), ii) a ketamine group (25 mg/kg/day ketamine, intraperitoneal injection)^9^, and iii) a ketamine/Lipotoxin group (ketamine plus intravesical instillation of Lipotoxin [0.8 ml] retained for 1-h on days 14 and 21, under 2–3% isoflurane anesthesia with mechanical ventilator). Lipotoxin was prepared as previously reported^28^: liposomes (Lipella, Pennsylvania) 10 mg/ml mixed with onabotulinumtoxinA (Allergan, California) 20 U/ml.

### Resting-state fMRI

Under intramuscular zoletil (50 mg/kg) injection anesthesia and bladder emptying with a polyethelence-50 catheter through the urethra, four rats in each group were used to evaluate the activation of PAG matter on days 0, 14, and 28. After emptying the bladder, the rats were prepared for MRI scanning less than 5 min elapsed. The fMRI was performed using a 9.4-T horizontal-bore animal MR scanning system (Biospec 94/20, Bruker, Ettingen, Germany). This scanning system comprises a self-shielded magnet with a 20 cm clear bore and a BGA-12S gradient insert (inner diameter: 12 cm) that offers a maximal gradient strength of 675mT/m with transmitter only coil and receiver rat brain surface array coil for signal detection from the head of the rat.

According to orientation of landmark structures from the sagittal images^21^, using multi-slice turbo rapid acquisition with refocusing echoes (Turbo-RARE) sequence with the following parameters: field of view (FOV) = 35.0 × 17.5 mm; matrix size = 256 × 128; spatial resolution = 137 × 137 μm; slice thickness = 0.5 mm; effective echo time (TE) = 30.0 ms; echo time = 10 ms; repetition time (TR) = 3000 ms; rare factor = 8; refocusing flip angle = 180 deg.; number of averages = 2; number of repetitions (NR) = 1; total acquisition time = 1 min 36 s. T2 -weighted axial anatomical reference imaging was performed on 15 adjacent slices from a restricted area of the brain that include the PAG using Turbo-RARE sequence acquisition with the following parameters: field of view (FOV) = 25.0 × 25.0 mm; matrix size = 256 × 256; spatial resolution = 98 × 98 μm; slice thickness = 1.0 mm; effective echo time (TE) = 30 ms; echo time = 10 ms; repetition time (TR) = 3000 ms; rare factor = 8; refocusing flip angle = 180 deg.; number of averages = 5; number of repetitions (NR) = 1; total acquisition time = 8 min. (Supplementary Figure S1).

By using identical spatial dimensions as in the T2-weighted axial reference imaging, we acquired functional images through the echo-planar imaging (EPI) sequence with the following parameters: field of view (FOV) = 25.0 × 25.0 mm; matrix size = 96 × 96; spatial resolution = 260 × 260 μm; slice thickness = 1.0 mm; effective spectral bandwidth = 250000 Hz; echo time (TE) = 20.0 ms; repetition time (TR) = 2000ms; segment = 1; number of averages = 1; total acquisition time = 4 min. One hundred twenty EPI volumes were acquired for each run. Rats were in resting state during all imaging sessions. Images were preprocessed using conventional procedures: registration to a segmented rat brain atlas and motion correction with SPM8 (Wellcome Department of Cognitive Neurology, London, UK), spatial smoothing (FWHM = 1 mm), regressions of motion parameters and white matter/ventricle signals, and band-pass filtering (0.002–0.1 Hz). Resting-state fMRI images were viewed as single intensity in the PAG region of the rats’ brains were referred to the standard anatomical atlas^29^ and analyzed with ImageJ 1.51. The procedures to localize the PAG area were shown in Supplementary Figure S1.

### Metabolic cage

On day 23, 12 rats in each group were placed in individual 3701M081 metabolic cage (Tecniplast, Buguggiate, Italy), as previously reported^30^. After a 24-h familiarization period, a known volume of water was filled in the animal drinking bottles. Both micturition frequency and urine output were determined using a cup fitted to an FT-104 force transducer (iWox/CB Sciences, Inc., Dover, NH, USA). The volume of liquid consumed and urine production were measured for 3 days. Urine samples were subjected to ketamine and norketamine assays through liquid chromatography-mass spectrometry.

### Cystometry

Rats were anesthetized with subcutaneous urethane (1.0 gm/kg) on day 28. A polyethelence-50 catheter was inserted in the urethra and was connected through a T-tube to a pressure transducer and a microinjection pump (infros AG, CH-4130, Bottmingen, Switzerland). Room temperature saline was infused into the bladder at a rate of 0.08 ml/ml and cystometrography was recorded using a Gould polygraph (RS3400; Gould, Cleveland, OH). After starting the saline infusion, we waited at least 30 min for voiding patterns to stabilize. Thus, reproducible micturition cycles were recorded for a 1-h period.

### Bladder morphology and immunohistochemical study

To characterize morphological changes in rats’ bladders, paraffin sections were subjected to routine hematoxylin and eosin staining and immunohistochemical analyses.

For immunohistochemical procedures, paraffin sections (4μm) of formalin-fixed bladders were dewaxed, rehydrated, and autoclaved. The sections were treated with 3% H_2_O_2_ to quench endogenous peroxidase. A primary antibody of ZO-1 (1: 500 dilution, Proteintech) or substance P (1: 1000 dilution, Abcam) was incubated with the sections. Secondary antibody conjugated with horseradish peroxidase was used. The slides were incubated in 3,3-diaminobenzidine, counterstained with Mayer’s hematoxylin (Novolink, RE7280-K), and mounted with malinol.

### Determination of protein levels in bladder mucosa and muscle layer

Bladder bodies were divided into mucosa and detrusor layers by a microdissection technique^31^. Interested detrusor inflammatory markers of KIC were investigated, namely IL-β, IL-6, TNF-α, Nerve growth factor, NF-κB and COX2^9,19,32^. Mucosal neuroceptors involved in the urothelial afferent signaling were evaluated, such as TRPV1, P2X_2_ and P2X_3_ receptors^33^. Also, the detrusor neuroceptors engaged in detrusor contraction were examined, such as M_2-_ and M_3_-mAchRs and P2X_1_ receptor^33^. The Western immunoblotting was performed as previously described^30^. In brief, alternative samples from each group were homogenized on ice in CelLytic^TM^MT cell lysis buffer (Sigma-Aldrich) containing a protease inhibitor. The total protein was measured using the Pierce 660-nm protein assay (Thermo, Waltham, MA, USA). Sodium dodecyl sulfate-polyacrylamide gel electrophoresis was performed using the Laemmli buffer system. Nuclear/Cytosol Fractionation Kit (BioVision, Inc., Milpitas, CA, USA) was used for preparing cytosolic and nuclear extracts and Western blot analysis of NF–κB translocation according to the manufacturer’s instructions.

Antibodies raised against SNAP25 (1: 1000 dilution; Cell signal), E-cadherin (1:1000 dilution; Cell Signal), nerve growth factor (1:200 dilution; Cell Signal), IL-1β (1:500 dilution; Abcam) IL-6 (1:1000 dilution; Abcam), TNF-α (1:250 dilution, Santa Cruz), NF-κB (1: 2500 dilution; Cell SiZgnal), COX-2 (1:200 dilution; Abcam), TRPV1 receptor (1:1000 dilution; Alomone), purinergic receptor P2X_1_ (1:10000 dilution; Alomone), purinergic receptor P2X_3_ (1: 1000 dilution; Alomone), M_2_-mAChR (1:1000 dilution; Alomone), M_3_-mAChR (1:1000 dilution; Alomone), and GAPDH (1: 10,000 dilution; Millipore) were used.

### Statistical Analysis

All data are presented as the mean ± standard error. Data were subjected to 1-way ANOVA and multiple comparisons by Dunnett’s test. For all statistical tests, p < 0.05 was considered significant.

### Data available statement

The datasets generated during and/or analyzed during the current study are available from the corresponding author on reasonable request.

## Electronic supplementary material

Supplementary Figures S1-S4
