# Supplementary material for: Potential Orphan Drug Therapy of Intravesical Liposomal Onabotulinumtoxin-A for Ketamine-Induced Cystitis by Mucosal Protection and Anti-inflammation in a Rat Model
Source: Sci Rep. 2018 Apr 11;8:5795. doi: 10.1038/s41598-018-24239-9 (PMC5895575; doi:10.1038/s41598-018-24239-9)

Potential Orphan Drug Therapy of Intravesical Liposomal Onabotulinumtoxin-A for Ketamine-Induced Cystitis by Mucosal Protection and Anti-inflammation in a Rat Model

Wei-Chia Lee, M.D. Ph.D. [1], Chia-Hao Su, Ph.D. [2], You-Lin Tain, M.D. Ph.D.[3], Cheng-Nan Tsai B.S. [1], Chun-Chieh Yu, M.S.[2], Yao-Chi Chuang, M.D. [1, 4]*

[1]Division of Urology, [2]Institute for Translational Research in Biomedicine, [3]Department of Pediatrics, and [4]Center for Shock Wave Medicine and Tissue Engineering, Kaohsiung Chang Gung Memorial Hospital and Chang Gung University College of Medicine, Kaohsiung, Taiwan.

Running title: Lipotoxin can ameliorate ketamine-induced cystitis in rats

***Address correspondence and reprint requests to**: Yao-Chi Chuang, MD. Division of Urology, Kaohsiung Chang Gung Memorial Hospital, 123 Ta Pei Rd., Niao Song Qu, Kaohsiung City, Taiwan, Republic of China, e-mail: chuang82@ms26.hinet.net, Tel. 886 7-7317123, Fax: 886-7-7318762.

Supplementary Figure S1

Procedures for magnetic resonance imaging (MRI) and the regions of interest (ROI) of periaqueductal grey (PAG) by diffusion tensor imaging (DTI). (a) Demarcation of the area of the brain (rectangular box) that was subjected for functional imaging. (B) Regions of interest (ROIs) placed manually over the PAG (referred to the standard anatomical atlas)[29] on the side for resting-state fMRI and analysis.

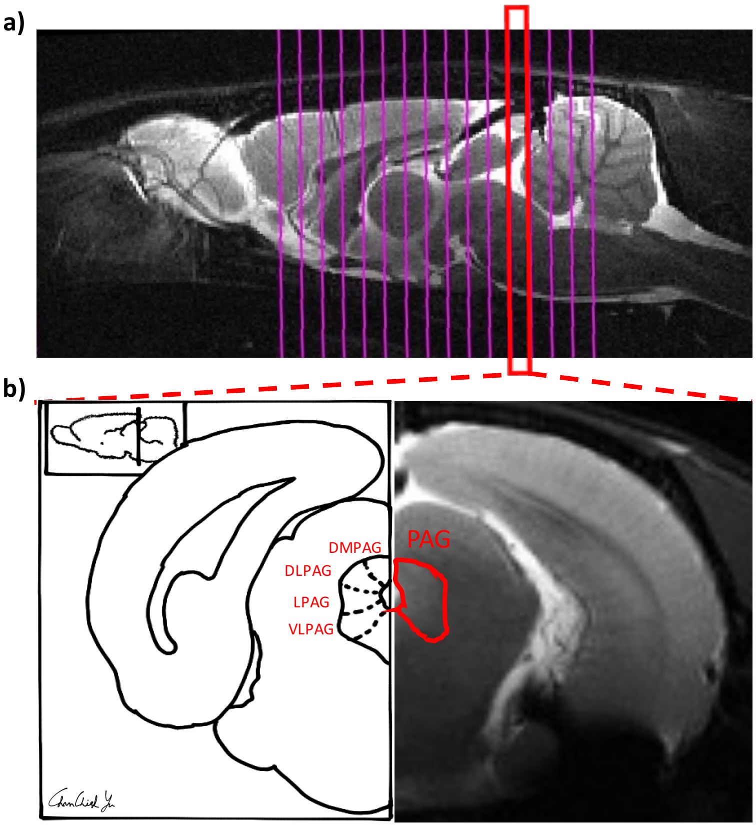

Supplementary Figure S2

Full-length blots/gels of bladder proteins illustrated in figure 2

D.

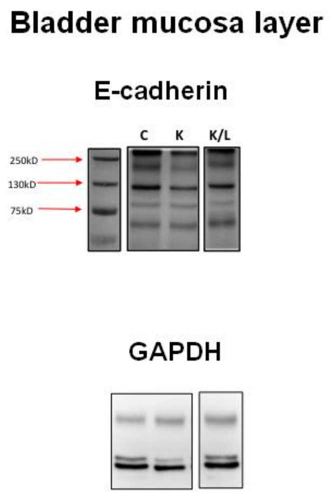

E.

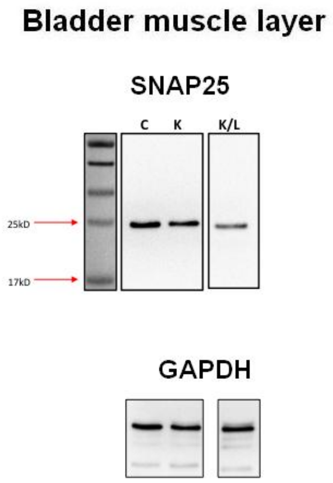

Supplementary Figure S3

Full-length blots/gels of bladder proteins illustrated in figure 3

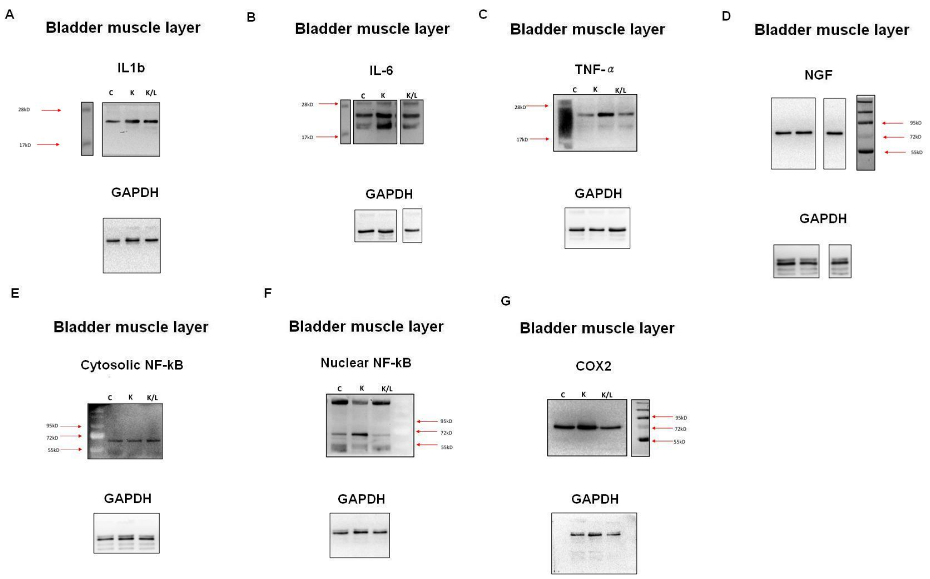

Supplementary Figure S4

Full-length blots/gels of bladder proteins illustrated in figure 4

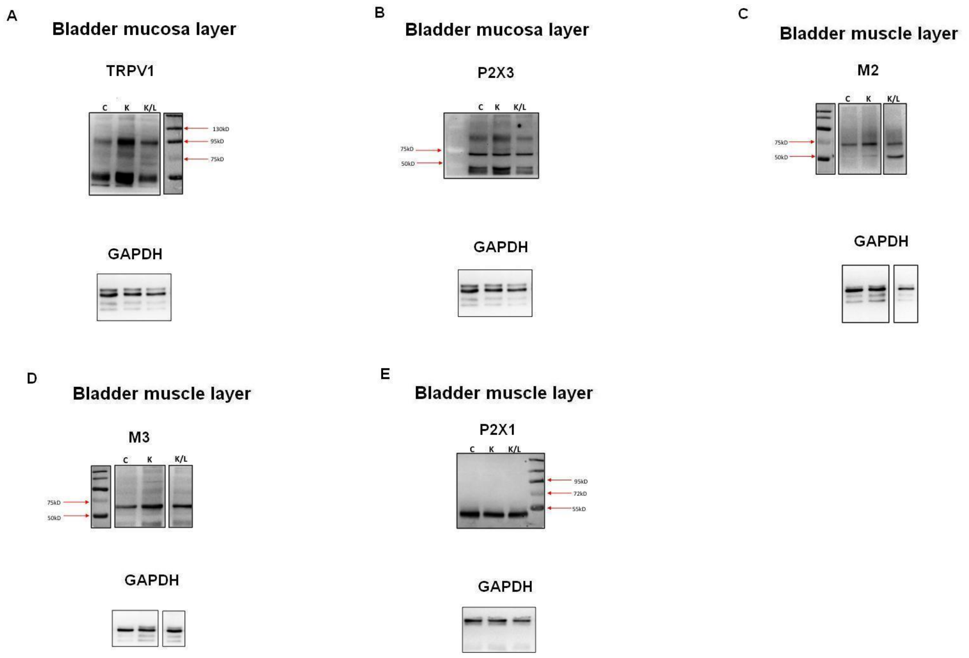

Supplement: Supplementary file 1 — Supplementary Figures S1-S4 [file 41598_2018_24239_MOESM1_ESM.pdf]
